# Genome-wide association and targeted analysis of copy number variants with psoriatic arthritis in German patients

**DOI:** 10.1186/s12881-017-0447-y

**Published:** 2017-08-23

**Authors:** Steffen Uebe, Maria Ehrlicher, Arif Bülent Ekici, Frank Behrens, Beate Böhm, Georg Homuth, Claudia Schurmann, Uwe Völker, Michael Jünger, Matthias Nauck, Henry Völzke, Heiko Traupe, Michael Krawczak, Harald Burkhardt, André Reis, Ulrike Hüffmeier

**Affiliations:** 1 Institute of Human Genetics, Friedrich-Alexander-Universität Erlangen-Nürnberg (FAU), Schwabachanlage 10, 91054 Erlangen, Germany; 2 Division of Rheumatology and IME Fraunhofer Project Group Translational Medicine & Pharmacology, Goethe University, Frankfurt/Main, Germany; 3 Interfaculty Institute for Genetics and Functional Genomics, University Medicine and Ernst-Moritz-Arndt University Greifswald, Greifswald, Germany; 4 Clinic of Dermatology, University of Greifswald, Greifswald, Germany; 5 Institute of Clinical Chemistry and Laboratory Medicine, University of Greifswald, Greifswald, Germany; 6 Institute for Community Medicine, University of Greifswald, Greifswald, Germany; 7 Department of Dermatology, University of Münster, Münster, Germany; 8 Institute for Medical Informatics and Statistics, Christian-Albrechts University Kiel, Kiel, Germany

**Keywords:** Copy number variant, Psoriatic arthritis, SNP arrays, Genome-wide association study, *TRB*, Psoriasis susceptibility loci

## Abstract

**Background:**

Psoriatic Arthritis (PsA) is a chronic inflammatory disease of the joints. PsA is etiologically complex, and 11 susceptibility loci have been identified so far. Most of these overlap with loci associated with psoriasis vulgaris (PsV), the most common psoriatic skin manifestation which is also frequently seen in PsA patients. In addition, two copy number variants (CNVs) are associated with PsV, one of which, located within the *LCE3* gene cluster, is also associated with PsA. Finally, an intergenic deletion has been reported as a PsA-specific CNV.

**Methods:**

We performed a genome-wide association study (GWAS) of CNVs in PsA and assessed the contribution to disease risk by CNVs at known psoriasis susceptibility loci.

**Results:**

After stringent quality assessment and validation of CNVs of the GWAS with an alternative quantitative method, two significantly associated CNVs remained, one near *UXS1*, the other one at the *TRB* locus. However, MLPA analysis did not confirm the CN state in ~1/3 of individuals, and an analysis of an independent case-control-study failed to confirm the initial associations. Furthermore, detailed PCR-based analysis of the sequence at *TRB* revealed the existence of a more complex genomic sequence most accurately represented by freeze hg18 which accordingly failed to confirm the hg19 sequence.

Only rare CNVs were detected at psoriasis susceptibility loci. At three of 12 susceptibility loci with CNVs (*CSMD1*, *IL12B*, *RYR2*), CN variability was confirmed independently by MLPA. Overall, the rate of CNV confirmation by MLPA was strongly dependent upon CNV type, CNV size and the number of array markers involved in a CNV.

**Conclusion:**

Although we identified PsA associations at several loci and confirmed that the common CNVs at these sites were real, ~1/3 of the common CNV states could not be reproduced. Furthermore, replication analysis failed to confirm the original association. Furthermore, SNP array-based analyses of CNVs were found to be more reliable for deletions than duplications, independent of the respective CNV allele frequency. CNVs are thus good candidate disease variants, while the methods to detect them should be applied cautiously and reproduced by an independent method.

**Electronic supplementary material:**

The online version of this article (doi:10.1186/s12881-017-0447-y) contains supplementary material, which is available to authorized users.

## Background

Psoriatic arthritis (PsA) is a common, chronic, inflammatory arthritis that is mainly observed in combination with the most common skin manifestation of psoriasis, namely psoriasis vulgaris (PsV). PsA is classified as a spondyloarthropathy and is usually sero-negative for auto-antibodies. Inflammation typically manifests at peripheral large and/ or small joints, the spine, and/ or entheses.

PsA is a complex disease, because it is known to be caused by a combination of different genetic and environmental risk factors. Interestingly, most recent heritability estimates suggest a 2- to 5-fold stronger genetic component in PsA than in PsV. Thus, the relative risk of first-degree family members (λ_1_) in PsA equals 30 to 39 [1, 2] compared to 7–19 in PsV [1, 3]. However, the number of identified genetic risk factors is much higher for PsV than for PsA because of the larger samples analyzed to date. Genome-wide association studies (GWAS) or more targeted analyses of immunologically relevant genes have accelerated the identification of susceptibility factors and 44 PsV susceptibility loci have been confirmed at a genome-wide level of significance so far [4–12]. The majority of genetic susceptibility factors for PsA have been shown to overlap with those of PsV [9, 13–16]. This notwithstanding, a non-coding variant at 5q31 overlapping with juvenile idiopathic arthritis, Crohn’s disease and asthma [14] and a missense variant in *PTPN22* [15] as well as the HLA-B27 locus may be more joint-specific risk factors.

Similar to other complex diseases, GWAS for PsV and PsA were focused upon single nucleotide polymorphisms (SNPs). Copy number variants (CNVs), however, are also known to contribute substantially to the diversity of the human genome [17, 18] and may thus be involved in the pathogenesis of both monogenic and common complex diseases as well. For PsV, an increased copy number at the highly polymorphic ß-defensin (*DEFB*) gene cluster at 8p23.1 has been shown to increase disease susceptibility [19, 20]. In addition, a frequent 32 kb deletion within the late cornified envelope 3 gene cluster (*LCE3C_3B-del*) was found to be a risk factor for both PsV and PsA [5, 21–24]. More recently an intergenic deletion has been shown to be associated with PsA, but not with PsV [25].

In view of the known associations between CNVs and psoriasis and to improve our understanding of the genetic basis of PsA, we performed a GWAS of CNVs for PsA, using previously published data from our own group of PsA patients [16].

## Methods

### Study groups and data

To investigate the association between PsA and CNVs at a genome-wide level, we used SNP array-derived data (Affymetrix Genome-Wide Human SNP Array 6.0) of 572 German patients with PsA [16] and of 4081 healthy individuals from a population-based control cohort (free of psoriasis) (Study of Health in Pomerania; SHIP) [26]. As described elsewhere [27], we excluded individuals with excessive numbers of CNVs, leaving a discovery cohort of 478 patients and 3798 controls. A primary component analysis based on SNP genotypes of 478 PsA patients and 3798 SHIP individuals was performed using smartpca from the EIGENSTRAT software suite [28]. We did see evidence for marginal stratification within the SHIP data set, but the vast majority of SHIP samples clustered with PsA cases in the first 6 eigenvectors, indicating no major influence of population stratification effects.

DNA samples from independent groups of PsA patients (*n* = 251) and healthy blood donors (*n* = 451), both of which represented subgroups of previously described cohorts [29], were used for the replication of the disease association of a deletion near *UXS1*. For the replication of the disease association of a CNV at *TRB*, we used DNA from 782 PsA patients, including all individuals of our discovery study, and 897 independent control individuals [30]. In order to avoid confusion, we henceforth use the term “replication” for a study that aims to confirm a disease association, whereas “validation” refers to the technical confirmation of a CNV by MLPA (see below).

### CNV genotyping and procedure of quality assessment in the GWAS

SNP array-based CNV genotyping analysis and quality assessment of CNVs were done as described in detail elsewhere [27]. Briefly, we used the CNV calling algorithm *Birdseye* to analyse the genome-wide data [31] and considered CNVs covered by ≥ 5 array-markers spanning ≥ 5000 base pairs (bp), thereby identifying 26 CNVs. Within those, we identified batch effects (differences in CNV frequencies between different groups of conjointly processed microarrays) as a major confounder and corrected for it with a linear mixed model analysis. In this latter analysis, association to 12 CNVs was not confirmed. Within the 26 CNVs, we observed further problematic issues that partly overlapped in single CNVs: association to CNVs’ boundary markers only (*n* = 4), no evidence for differences in CNV frequency when compared to one or both of two smaller control groups (*n* = 11), location in regions of high and/ or extended sequence homology (*n* = 18). A correlation analysis provided evidence that certain characteristics of the CNVs as well as problematic issues were not independent from each other. Problematic issues were more common in the 12 CNVs with no confirmation of association in the linear mixed model.

**Fig. 1:**
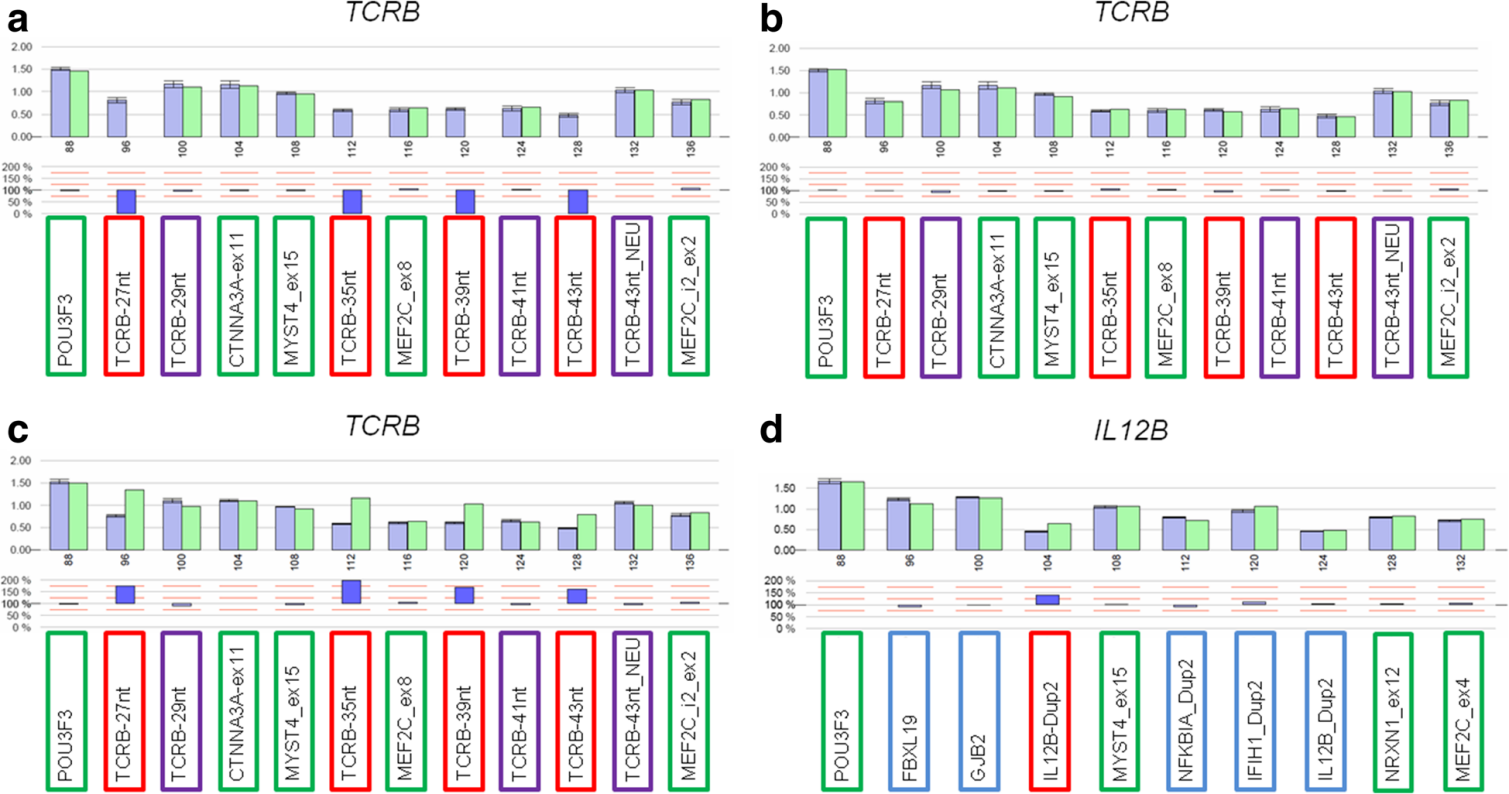
Investigation of copy number states by MLPA analysis. **a-c**: Copy number (CN) states at *TRB*, calibrated by carriers of one gene copy. The CNV region was targeted by four different MLPA probes (TCRB_27nt, TCRB_35nt, TCRB_39nt and TCRB_43nt; *red* frame), CN-stable regions encompassing the deleted region were covered by three additional MLPA probes (TCRB_29nt, TCRB_41nt and TCRB_45nt_NEU; *purple* frame). Five CN-stable control loci from other genomic regions (*green* frame) were included in the analysis as well (*red* and *purple* coloring corresponds to the respective probes in Fig. 2f). **a** Deletion in homozygous form, **b** Deletion in heterozygous form and **c** no deletion, i.e. two gene copies; **d** CN states at a CNV at the *IL12B* locus, calibrated by carriers of two gene copies; duplication targeted by one MLPA probe (IL12B_Dup2; red frame). Signals for four control loci located at CN-stable regions (*green* frame) and five CN variable regions (*blue* frame) were included in the analysis as well (data are from different PsA patients; one at *IL12B* [Dup1, not overlapping with Dup2], *FBXL19*, *GJB2, NFKBIA* and *IFIH1*, respectively)

Mainly within the 14 CNVs that passed the linear mixed model and provided less/ no evidence for the further problematic issues, we selected 11 CNVs for validation by an independent quantitative method, namely multiplex ligation-dependent probe amplification (MLPA). For 8 CNVs, probe pairs could be self-designed as described [27, 32], and MLPA was performed as recommended by the manufacturer (MRC Holland, Amsterdam, The Netherlands) and as described in more detail elsewhere [27, 32]; in case of three CNVs, a probe design was impossible. Figure 1 exemplifies the analysis of relevant CNVs. At least five control individuals with two gene copies at all reference loci (one gene copy in the case of the *TRB* deletion) were included in each experiment as internal controls. Every target CNV was covered by at least one pair of CNV-specific MLPA probes that captured the most common CNV at the respective locus. Individuals with one of the three CN states (wild-type (WT)/ WT; WT/ CNV, CNV/ CNV) were re-analysed by MLPA. Since no DNA was available for the SHIP controls, however, their CNV genotypes could not be confirmed by MLPA.

CNVs at two loci passed both quality assessment and validation with MLPA. These CNVs either neighboured *UXS1* or were located at the *TRB* locus (size and position in different genomic annotations as indicated in Additional file 1: Table S1).

### Molecular analyses at the *TRB* locus

**Fig. 2:**
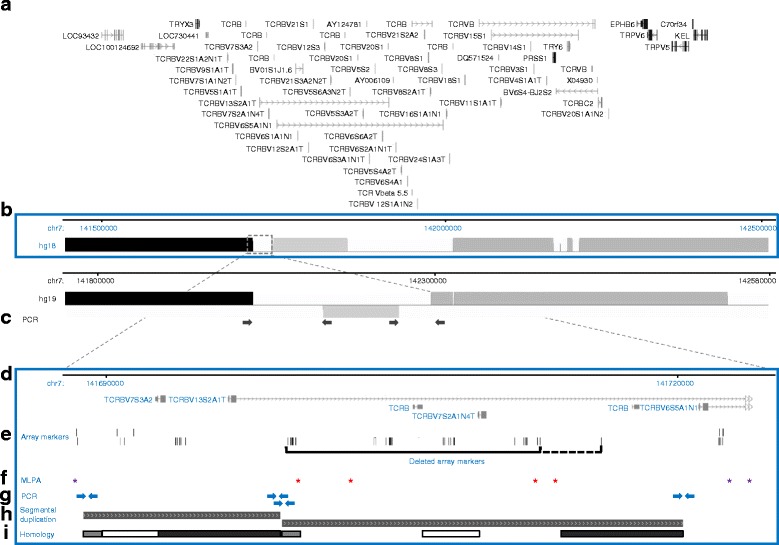
Genomic annotation of the *TRB* gene cluster on chromosome 7q34. **a** Annotated UCSC genes in NCBI36/hg18 (genome-euro.ucsc.edu); **b** Sequence alignments of genome freezes NCBI36/hg18 and GRCh37/hg19 with chromosomal positions, similar or identical sequences (*grey* and *black* bars, respectively), unique/ unaligned sequences (*white*), sequences identified to be inverted in freeze hg19 are given below the baseline; **c** PCR products specific for inversion GRCh37/hg19 (*arrows*); **d-i** Magnification (*dotted box*/ *lines*) of genome freeze NCBI36/hg18; **d** Genomic position of frequent *TRB* deletion, chromosomal position and UCSC genes; **e** Positions of array-markers: *upper* row = SNP markers; *lower* row = CN markers; *solid black line* = most common, minimal size of deletion (13,136 bp or 28 markers). The *dashed line* indicates one additional array-marker that is commonly deleted as well (deletion of 16,338 bp or 29 markers); **f** Positions of pairs of MLPA probes (asterisks) encompassing the *TRB* locus. Probes marked in *purple* encompass the CNV, probes marked in red are located within the deletion (see Fig. 1a-c); **g** *Arrows* indicate PCR primers for amplicons specific to NCBI36/hg18 (*arrows*); **h** Segmental duplication of 90–98% similarity (*grey* patterned bars); **i** Homologous sequences in the first and second part of the segmental duplication (indicated by bar of same color)

Since most of the array markers spanning the CNV at the *TRB* gene were only present in the hg18 human genome assembly, but not in hg19, we analysed this locus both in silico and experimentally. To this end, we aligned hg18 and hg19 using the Mauve software (http://darlinglab.org/mauve/), and performed additional laboratory experiments to infer which of the two assemblies was more reliable at *TRB*. We established four different PCRs with oligonucleotides specific to the sequence in hg18 and two PCRs specific to hg19 (Fig. 2). PCR and Sanger sequencing following standard protocols [29] were performed in Eppendorf Mastercyclers (Eppendorf, Hamburg, Germany). Purification of PCR products (Agencourt AMPure kit) and sequencing reactions (CleanSEQ) were performed using the Beckman-Coulter Biomek Nxp robotic system (Beckman-Coulter, Krefeld, Germany). MLPA identified one of the three possible CN states (0, 1, 2 copies) at the *TRB* locus in 135 of individuals. For 10 of these individuals (5 with 0 copies, 3 with 1 copy, and 2 with 2 copies), however, PCR failed to yield products specific for genome freeze hg19 at all, whereas DNA of 19 individuals (5 with no copies, 11 with 1 copy and 3 with 2 copies) yielded PCR products specific to hg18. All sequences were analysed using SeqMan software (DNASTAR, Madison, USA) and were aligned to the respective reference sequences (NCBI36/hg18 and GRCh37/hg19).

### Analysis of CNVs at psoriasis susceptibility loci

In addition to a GWAS, we analysed CNVs at 30 psoriasis disease loci of at least borderline genome-wide significance published until November 2010 [4–11, 13, 16]. First, we investigated the degree of linkage disequilibrium (LD) in our GWAS data [16] between the most significantly disease-associated SNP at each locus and the surrounding SNPs, using HaploView 4.2 (https://www.broadinstitute.org/haploview/haploview). Regions of relevant LD with the most significantly associated SNP were defined either as previously described [33] or by having an r^2^ > 0.1. In a second step, CN data of PsA patients were screened for CNVs overlapping these LD regions as described elsewhere [27]. In order to validate the disease association of CNVs in the overlapping regions, an MLPA probe was designed for each disease locus at which CNVs were identified in our PsA cohort (feasible for all but one locus). The probes were found to cover the majority of the observed CNVs (Additional file 1: Table S2). A subset of 7–14 PsA patients including carriers of the rare CNV were included in the validation step by MLPA.

### Additional statistical analyses

**Fig. 3:**
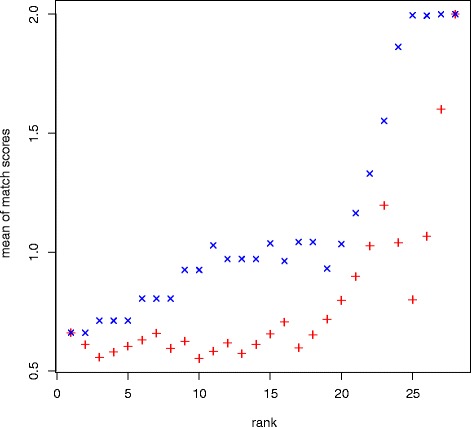
Correlation between degree of copy number validation and median size and marker count of CNVs. X axis: ranking of 28 CNVs (8 frequent, 20 low-frequency) according to median size (*red vertical crosses*) or marker count (*blue diagonal crosses*); Y axis: mean of mean match scores for this CNV and all CNVs with a higher size or marker count

For each CNV detected in our study (*n* = 28; Tables 1, 2, 3, Additional file 1: Table S2), MLPA results were compared to the genotypes determined in the GWAS [27]. To this end, we defined a CNV-specific match score for each individual that was set equal to 2, if both genotypes were found to be of the same type and copy number; a match score of 1 was assigned, if the two genotypes matched only in type (i.e. deletion or duplication), but not in copy number; otherwise, the match score was set to 0. Next, the mean match score taken over all individuals was determined for each CNV. To assess the influence of the CNV type (deletion or duplication) on the degree of MLPA validation, we compared the match score distributions obtained for the two types of CNVs using a Mann-Whitney rank sum test. To assess the influence of the segment size and number of array-markers involved in a CNV, we first determined the median segment size and median marker count for each of the 28 CNVs. Then, the mean was calculated over the mean match scores of all loci with a median size or median marker count larger than the CNV-specific value (Fig. 3). Finally, Spearman’s rank correlation coefficient ρ was calculated as a correlation coefficient between those means and the median CNV size or marker count, respectively.

**Table 1:** Allele counts (frequencies) of the risk/ non-risk alleles [n (%)] of the CNV near *UXS1*

absolute chromosomal position (hg18)	relative position to nearest gene(s)	Part of study	n (%) –risk allele in PsA	n (%) – non-risk allele in PsA	n (%) –risk allele in Ctrl	n (%) – non-risk allele in Ctrl	*P*-value	OR [95% CI]
chr2: 106,246,527–106,251,789	69 kb upstream of *UXS1*	discovery^a^	861 (90.1)	95 (9.9)*	6026 (79.3)	1570 (20.7)*	2.84 × 10^−15^	2.36 [1.90–2.94]
		unfiltered discovery^b^	635 (66.4)	321 (33.6)	5145 (67.7)	2451 (32.3)	0.44	0.94 [0.82–1.09]
		replication	331 (66.5)	167 (33.5)*	594 (66.0)	306 (34.0)*	0.94	1.02 [0.81–1.29]

Deletion is marked by *. The *p*-value corresponds to a χ2 test of 478 PsA patients and 3798 control individuals. Odds ratios [95% confidence interval] of the discovery study (array-based analysis) were calculated for the same cohorts. The independent replication cohorts (MLPA-based analysis) comprised 251 patients and 451 control individuals. ^a^Discovery describes the initial SNP array-based analysis with the filter criteria of 5 markers and 5 kb before validation, ^b^unfiltered discovery the number of CNVs and wildtype-alleles in the initial SNP array-based dataset that was unfiltered for no. of markers and for size, as analyzed after validation with MLPA

**Table 2:** Allele counts (frequencies) of CNV at *TRB* in PsA patients and controls (Ctrl)

part of study	n (%) –risk allele in PsA	n (%) –non-risk allele in PsA	n (%) –risk allele in Ctrl	n (%) – non-risk allele in Ctrl	*P*-value	OR [95% CI]
discovery^a^	776 (81.2)	180 (18.8)*	5288 (69.6)	2308 (30.4)*	1.22 × 10^−13^	1.88 [1.59–2.23]
extended PsA, independent Ctrl cohort	801 (51.1)	765 (48.9)*	947 (52.8)	847 (47.2)*	0.34	0.94 [0.82–1.07]

Deletion is marked by *. The *p*-value corresponds to a χ2 test of of 478 patients and 3798 control individuals. Odds ratio [95% confidence interval] for the discovery study (array-based analysis) were calculated for the same cohorts. The replication cohort (MLPA-based results) was partially overlapping for PsA, and consisted of 782 patients and 897 controls. ^a^Discovery describes the initial SNP array-based analysis with the filter criteria of 5 markers and 5 kb before validation with MLPA

**Table 3:** Low-frequency CNVs at psoriasis susceptibility loci in PsA patients (*n* = 478) and controls (*n* = 3798) (Ctrl)

nearest gene/ locus	type of aberration	no. of CNV alleles (PsA)	frequency in % (PsA)	validation (no. of PsA patients analyzed by MLPA)	no. of CNV alleles (Ctrl)	frequency in % (Ctrl)	*p*-value
*CSMD1*	deletion	1	0.10	validated (7)	7	0.09	1
*IL12B*	duplication	2	0.21	validated (14)	3	0.04	0.1
*RYR2*	deletion	2	0.21	validated (7)	12	0.16	0.7
*ERAP1*	deletion	2	0.21	not validated (7)	1	0.01	n/a
*ERAP1*	duplication	1	0.10	not validated (7)	4	0.05	n/a
*FBXL19*	deletion	1	0.10	not validated (14)	0	0	n/a
*FBXL19*	duplication	2	0.21	not validated (14)	22	0.33	n/a
*GJB2*	duplication	1	0.10	not validated (14)	1	0.01	n/a
*IFIH1*	duplication	3	0.31	not validated (14)	6	0.09	n/a
*IL23A*	duplication	4	0.42	not validated (7)	8	0.12	n/a
*NFKBIA*	duplication	2	0.21	not validated (14)	6	0.09	n/a
*RNF114*	duplication	2	0.31	not validated (12)	7	0.09	n/a
*TNIP1*	duplication	1	0.10	not validated (7)	0	0	n/a
*TRAF3IP2*	duplication	3	0.31	not validated (12)	7	0.09	n/a

Gene nearest to the susceptibility locus, the type of aberration, the absolute no. of CNV alleles, their overall frequency in percent, the result of a validation by MLPA (in PsA patients only) and the results of a Fisher’s exact test (n/a: not applicable due to lack of validation) are given

## Results

In the GWAS, we observed an association with PsA of the CNVs near *UXS1* and at the *TRB* locus (Tables 1 and 2). The respective copy number states at these loci were confirmed by MLPA (Fig. 1a-c).

### CNVs at the *UXS1* locus

One of the disease associated CNVs was intergenic, located 69.3 kb upstream of *UXS1* on one side and 113.2 kb upstream of *PLGLA* on the other side. The *UXS1* gene encodes UDP-glucuronate decarboxylase, an enzyme that catalyses the formation of UDP-xylose from UDP-glucuronate used in the biosynthesis of glucosaminoglycans. *PLGLA* is a pseudogene of the gene encoding plasminogen, a major dissolvent of fibrin. The *UXS1* and *PLGLA* genes are positional candidates for PsA causality, especially *UXS1*, although we cannot exclude that other genes in the vicinity might also be affected by the deletion. Our association findings were not replicated in an independent sample of 251 PsA patients and 451 controls (Table 1). Although we could confirm the deletion and wild-type states in 85 of 135 PsA patients genotyped with both a SNP array and MLPA, discordant CN states emerged for 50 of 135 individuals (37.0%). We therefore re-analysed the SNP-array based CN data as described elsewhere [27] and identified smaller CNVs that had been excluded because one filter criterion for exclusion was a minimal size of 5000 bp.

When the frequent, albeit short CNVs were included in the initial association study, equal allele frequencies of the deletion were observed in PsA patients and control individuals (33.6% vs. 32.3%).

### Deletion at the *TRB* locus

The *TRB* locus encodes a T cell receptor, a molecule responsible for recognizing foreign antigens. Therefore, the frequent deletion detected at the *TRB* locus was recognized as a most interesting candidate for PsA causality. Comparison of the SNP array-based MLPA-based genotypes revealed discrepancies for 48 of 135 individuals (35.6%). For this CNV, we could exclude that smaller frequent CNVs - that escaped due to our filter criterion of a minimal size of 5000 bp as described in case of the CNV near *UXS1* - caused a false positive association. We decided to analyse this CNV in more detail. Seven different pairs of MLPA probes located either within the deletion or flanking it (hg18 assembly) (Fig. 2f) were used for genotyping and similar results were obtained with both types of probes (same coloring of target probes in Fig. 1a-c and Fig. 2f).

Sequence analysis and genomic alignment revealed different annotations in human genome freezes hg18 and hg19 (Fig. 2b). Thus, SNP array-markers identified to be disease-associated in our genome-wide study were not annotated in hg19, and a segmental duplication within the region was annotated only in hg18 (Fig. 2b, h). In addition, an inversion of a 118 kb was only present in hg19 (Fig. 2b). To investigate the differences in genomic annotation experimentally, we designed PCR products spanning the region of interest that were specific to either hg19 or hg18 (Fig. 2c, g). Using doubly genotyped PsA patients (i.e. genotyped with both the SNP array and MLPA) as a reference, we also genotyped control individuals by MLPA. Representative carriers of the different copy number states were analysed further by specific amplification and sequencing of PCR products from the *TRB* region. All three PCR products specific to the hg18 freeze, but not overlapping the deletion, could be amplified in individuals of all three copy number states. By contrast, the product from within the *TRB* deletion was absent in all homozygous deletion carriers (Fig. 2e, g), as was to be expected. In the hg19 freeze, no explicit sequence (“NNNNNN”) was annotated between the sequences that were present in both assemblies in the human genome (represented as black or grey boxes in Fig. 2b). Therefore, we designed PCR products specific to the hg19 freeze spanning these regions (Fig. 2c). However, we could not amplify any specific products in individuals of any copy number state, thereby providing evidence in favour of the hg18 assembly being closer to the actual sequence at this locus than hg19.

The 33 of 48 PsA samples with discordant SNP array-based and MLPA-based genotypes were reanalysed by PCR specific to the hg18 assembly. We identified 12 deletion homozygous patients through the lack of the PCR product. These genotypes were in accordance with MLPA, but not with the SNP array. Since PCR-based analysis could not distinguish between 1 or 2 copies, we could not evaluate the CN states of the remaining 23 samples. All of them showed either 1 or 2 copies when genotyped by MLPA. When we re-analysed the complete PsA cohort of the discovery study with MLPA, a similarly high discordance rate emerged between array and MLPA analysis (157 of 446 samples; 35.2%) as in the initially analysed subset of patients.

Since genotyping of the CNV appeared to be more reliable with MLPA than array-based data, we screened independent cohorts of 304 patients and 897 healthy controls with MLPA. When comparing the allele frequencies of all patients (*n* = 782) to those in independent control individuals (*n* = 897), no significant difference was observed (Table 2). Frequencies observed in the MLPA-based second analysis were significantly different from those of the discovery study (Table 2), suggesting difficulties in genotyping this CNV by array-based analyses.

### CNVs at psoriasis susceptibility loci

Of note, we did not detect association to the CNVs at previously identified copy number loci such as *LCE3C*/ *LCE3B* [5], the ß-defensin-cluster [19, 20] and the intergenic deletion [25]. As we had not observed association to the *LCE3C*/ *LCE3B* in a largely overlapping cohort of 650 German PsA patients [34], we did not expect to find evidence for association. Furthermore, the SNP array-based analysis could not adequately and reliably disentangle the multivariable ß-defensin cluster. Last but not least, the minor allele frequency of the intergenic CNV was similar in cases (8%) and controls (7.5%). More detailed results on the *LCE3* cluster and the ß-defensin cluster can be read elsewhere [27].

By using the SNP array-based approach to detect frequent CNVs at 30 previously identified susceptibility loci for PsV or PsA [4–11, 13, 16], we did not detect any. Therefore, CNVs at these loci do not seem to be reasonable candidate variants for disease-causality, at least not in German patients with PsA, but we have to consider the limitations of our method with regard to smaller sizes of (< 5000 bp) and the detection rate of CNVs using the procedure of SNP array-based analyses. One frequent deletion was identified at the *HLA-C* locus, but owing the high level of sequence homology (> 99%) to the *HLA-B* locus, we were unable to technically validate this CNV by MLPA. Therefore, it remained unclear whether the disease association of this locus was genuine or represented a technical artefact.

We exclusively identified low-frequency CNVs (MAF < 5%) at 12 of the 30 psoriasis susceptibility loci studied, with duplications (*n* = 16) being more frequent than deletions (*n* = 4) (Table 3). Only four different CNVs were confirmed by MLPA, namely two intronic deletions in the *RYR2*and *CSMD1* genes and two duplications at the *IL12B* locus. In case of CNVs at the remaining susceptibility loci, no copy number variability could be confirmed. We also observed low-frequency CNVs at the three loci in control individuals, but no evidence for disease association was apparent (Table 3).

### CNV characteristics and MLPA validation

We performed MLPA for eight frequent CNVs identified through GWAS (see Tables 1 and 2; [27]) and for 20 low-frequency CNVs at psoriasis susceptibility loci (Table 3; Additional file 1: Table S2) and compared the outcome to CNV calling from SNP array data (frequent CNVs [27]; low-frequency CNVs: Table 3; Additional file 1: Table S2). A significant correlation was observed between the type of aberration (deletion versus duplication) and the chance of MLPA validation (*p* = 3.01 × 10^−3^). Deletions were validated more often (8 of 11, 73%) than duplications (2 of 17, 12%), as is also reflected by the difference in the mean of mean match scores (1.32 for deletions; 0.24 for duplications).

We also observed evidence for a positive correlation between the mean match score of CNVs and its median segment size (Spearman’s ρ = 0.77) and an almost perfect correlation between the mean match score and the median marker count (Spearman’s ρ = 0.96; Fig. 2). Although larger CNVs comprising higher marker counts showed a higher level of concordance between SNP array-based genotype and MLPA-based genotype, a lower marker count or a smaller size of a CNV did not indicate discordance between SNP array-based and MLPA-based genotypes in case of all small CNVs.

## Discussion

Although CNVs have been shown to potentially play a role in the genetic basis of both PsV and PsA [5, 19, 20, 22, 25], our GWAS of CNVs failed to identify any novel disease-associated CNV for PsA. As a result of our three-stage analysis comprising thorough quality assessment [27], validation by MLPA as a second quantitative method and replication analysis in an enlarged case-control study, none of the originally identified disease-associations of CNVs remained significant. In view of previous experience with small CNVs in the context of immunological diseases (e.g. type 2 diabetes, Crohn’s disease and rheumatoid arthritis [35]), we chose to screen CNVs of 5000 bp or more. This notwithstanding, even if we considered small CNVs, no robust association between PsA and a frequent CNV could be found.

Previous studies reported on lower recovery rates and pronounced difficulties in genotyping frequent CNVs [36, 37], especially in the case of full gene deletions. The same reasons responsible in these instances may also explain the discrepancies in CNV genotyping between SNP array and MLPA noted in our study for the frequent deletion at the *TRB* locus.

Our analysis revealed differences in annotation of the *TRB* locus on chromosome 7q34 between genome freezes hg18 and hg19, and experimental evidence was generated in favour of hg18 being closer to the true DNA sequence. Through intensive investigation of this locus, we could validate a frequent CNV at *TRB*, but its initial association with PsA, probably due to wrong genotypes derived for the SNP array-based analysis could not be replicated. The locus on chromosome 7q34 is complex and comprises numerous annotated *UCSC* genes and transcript variants. T cell receptors are known to undergo somatic rearrangements of variable (V), diverse (D) and joining (J) gene segment/ chains (= V(D)J recombination). Moreover, an enormous variability in terms of the clonality of T-cell populations exists between individuals and increases the complexity of this genomic locus [38, 39]. In summary, high variability and lack of representativeness of the reference sequences might have contributed notably to the difficulties in SNP array-based genotyping of the *TRB* cluster.

We failed to identify association of PsA with a novel CNV. This might be due to the limited power that we had in 478 patients, while we cannot exclude that we missed associations to CNVs due to the limitations of the SNP array-based analysis.

Our study strongly suggests that independent validation of CNVs is essential for their use as disease markers, particularly because the low-frequency CNVs considered at psoriasis susceptibility loci in our study were characterized by an unacceptably low level of validation. Low-frequency duplications were remarkably more difficult to validate than low-frequency deletions. Moreover, because no frequent duplication was validated at all, we assume that the detection of duplications is generally more complicated and less reliable than that of deletions. These findings are in accordance with previous findings, for example of Zhang et al. [36] who also reported lower recovery rates for duplications. In addition, recovery rates for computer-simulated duplications were found to be lower than those for equally sized deletions [40].

We observed a robust correlation between both the number of markers involved and the size of CNVs on the one hand and the level at which these CNVs are successfully validated. The impact of marker count and CNV size on the reliability of CNV calling has been discussed before [36, 39, 41] and was quantified by simulation [40], indicating that recovery rates of CNVs increase when the marker count increases. This increase was also shown to be more rapid for deletions than for duplications.

## Conclusions

Although we were able to detect an initial PsA association for two CNVs and could confirm that the frequent CNVs involved were real, we failed to validate the copy number states in a significant number of cases. Also, we were unable to replicate the original disease association. Furthermore, SNP array-based analyses of CNVs are more reliable in pinpointing deletions than duplications, independent of the CNV allele frequency. Frequent CNVs remain good candidate variants for disease association, while we suggest to use also other methods to detect CNVs cautiously and to reproduce CNVs with an independent method. Other than SNP array-based analyses, e.g. digital PCR, might be more useful to detect CNVs.

## Additional file

Additional file 1: Tables S1 and **S2** are presented in the Supplementary data file. (DOCX 25 kb)

## Abbreviations

CNVs: Copy number variants
Ctrl: Control individuals
*DEFB*: Beta-defensins
del: Deletion
dup: Duplication
GWAS: Genome-wide association study/ ies
*LCE3*: Late cornified envelope group 3
LD: Linkage disequilibrium
MLPA: Multiplex ligation-dependent probe amplification
no.: Number
PsA: Psoriatic arthritis
PsV: Psoriasis vulgaris
SNPs: Single nucleotide polymorphisms
*TRB*: T cell receptor beta locus
WT: Wild-type
