# Supplementary material for: Genome-wide association and targeted analysis of copy number variants with psoriatic arthritis in German patients
Source: BMC Med Genet. 2017 Aug 23;18:92. doi: 10.1186/s12881-017-0447-y (PMC5569473; doi:10.1186/s12881-017-0447-y)
Supplement: Additional file 1: Tables S1 — and S2 are presented in the Supplementary data file. (DOCX 25 kb) [file 12881_2017_447_MOESM1_ESM.docx]

**Additional file**

**Genome-wide association and targeted analysis of copy number variants with psoriatic arthritis in German patients**

Steffen Uebe ^1^, Maria Ehrlicher ^1^, Arif Bülent Ekici ^1^, Frank Behrens ^2^, Beate Böhm ^2^, Georg Homuth ^3^, Claudia Schurmann ^3^, Uwe Völker ^3^, Michael Jünger ^4^, Matthias Nauck ^5^, Henry Völzke ^6^, Heiko Traupe ^7^, Michael Krawczak ^8^, Harald Burkhardt ^2^, André Reis ^1^, Ulrike Hüffmeier ^1^

**Additional file 1: Table S1: Size and positions (in base pairs) of most common deletions near *UXS1* and within *TRB* locus for different genome versions**

|  | ***UXS1*** | ***TRB*** |
| --- | --- | --- |
| Size in hg18 | 5,262 | 13,136 |
| hg18 | chr2:106,246,527-106,251,789 | chr7:141,699,450-141,712,586 |
| hg19 | chr2:106,880,095-106,885,357 | multiple alignments between  chr7: 142,002,087,087-142,252,627 |
| hg38 | chr2:106,263,639-106,268,901 | chr7 (KI270803v1_alt):  314919-328052; multiple alignments  between chr7:142,302,264-142421089 |

**Additional file 1 Table S2: Characteristics of CNVs used in correlation analysis.**

Indicated are **A** the nearest gene/ locus of the CNV, **B** its category of frequency (frequent >5%, low-frequent <5%), **C** the type of aberration, **D** no. of absolute observations (no. of observations covered by MLPA in case of frequent CNVs), **E** no. of different CNVs observed (covered by MLPA), **F** (the range of) minimal sizes of CNVs (in base-pairs), in case of two different CNV sizes, separation by “/”) and **G** the range of/ minimal no. of involved array markers identified in > 1 individual.

| **A** | **B** | **C** | **D** | **E** | **F** | **G** |
| --- | --- | --- | --- | --- | --- | --- |
| **nearest gene/ locus** | **frequency** | **type of aberration** | **no. of observations in PsA (no. covered by MLPA)** | **no. of different CNVs (covered by MLPA)** | **(range of) CNV size (bp)** | **range of/ no. of involved markers** |
| *CD109* | > 5% | del | 194/ (193) | 3 (2) | 8,439 – 9,552 | 8 - 11 |
| *OR51AA4* / *OR51A2* | > 5% | del | 299 (299) | 48 (48) | 5,077 –9,489 | 25 - 45 |
| *SIRPB1* | > 5% | del | 365 (365) | 22 (22) | 10,072 – 41,146 | 13 - 70 |
| *TRB* | > 5% | del | 161 (161) | 13 (13) | 5,043 – 22,207 | 13 - 35 |
| *UXS1* | > 5% | del | 93 (93) | 5 (5) | 5,262 – 7,313 | 12 - 14 |
| *TMPRSS11E* | > 5% | del | 284 (280) | 7 (6) | 5,937 – 39,729 | 5 - 11 |
| *FSTL5* | > 5% | dup | 227 (194) | 40 (30) | 5,344 – 67,096 | 6 - 84 |
| *HLA-DRB5* | > 5% | del | 202 (182) | 8 (7) | 43,081 – 117,564 | 12 - 34 |
| *CSMD1* | < 5% | del | 1 | 1 (1) | 37983 | 53 |
| *IL12B_1* | < 5% | dup | 1 | 1 (1) | 20119 | 13 |
| *IL12B_2* | < 5% | dup | 1 | 1 (1) | 627335 | 429 |
| *RYR2* | < 5% | del | 2 | 1 (1) | 5135 | 15 |
| *ERAP1_1* | < 5% | del | 2 | 2 (1) | 8099/ 25378 | 9/ 21 |
| *ERAP1_1* | < 5% | dup | 1 | 1 (1) | 21392 | 17 |
| *FBXL19_1* | < 5% | del | 2 | 2 (1) | 21619/ 22632 | 7/ 10 |
| *FBXL19_1* | < 5% | dup | 10 | 10 (2) | 11810 - 65798 | 5 - 22 |
| *GJB2* | < 5% | dup | 1 | 1 (1) | 15874 | 8 |
| *IFIH1_1* | < 5% | dup | 1 | 1 (1) | 8341 | 6 |
| *IFIH1_2* | < 5% | dup | 2 | 2 (2) | 15232/ 49137 | 10/ 30 |
| *IL23A* | < 5% | dup | 4 | 4 (1) | 5303 - 29301 | 7 - 26 |
| *NFKBIA_1* | < 5% | dup | 1 | 1 (1) | 21468 | 7 |
| *NFKBIA_2* | < 5% | dup | 1 | 1 (1) | 43362 | 26 |
| *RNF114_1* | < 5% | dup | 1 | 1 (1) | 5199 | 6 |
| *RNF114_2* | < 5% | dup | 1 | 1 (1) | 11020 | 5 |
| *TNIP1* | < 5% | dup | 1 | 1 (1) | 6043 | 7 |
| *TRAF3IP2_1* | < 5% | dup | 1 | 1 (1) | 6126 | 6 |
| *TRAF3IP2_2* | < 5% | dup | 1 | 1 (1) | 12694 | 11 |
| *TRAF3IP2_3* | < 5% | dup | 1 | 1 (1) | 13333 | 11 |
